# Applying advanced psychometrics to antipsychotic treatment trials: A protocol comparing effect estimates

**DOI:** 10.12688/openreseurope.23623.1

**Published:** 2026-06-17

**Authors:** Timo Schurr, Fiona Boland, David Byrne, Frank Doyle

**Affiliations:** 1 Department of Health Psychology, School of Population Health, RCSI University of Medicine and Health Sciences, Dublin, Ireland; 2 Data Science Centre, School of Population Health, RCSI University of Medicine and Health Sciences, Dublin, Ireland

**Keywords:** schizophrenia, antipsychotics, panss, bprs, effect size, psychometrics, confirmatory factor analysis, item response theory, network analysis, individual participant data

## Abstract

**Background:**

Antipsychotic trials in schizophrenia quantify treatment efficacy using clinician-rated scales such as the Positive and Negative Syndrome Scale (PANSS) and Brief Psychiatric Rating Scale (BPRS). If these scales include redundant or weakly informative items, measurement noise can blur drug-comparator differences that may dilute real effect sizes. Modern psychometric methods can refine and re-weight symptom scores that may improve precision and potentially increase detectable treatment effects, although it is unclear which approaches might be best.

**Aims:**

To 1) determine best psychometric models for the PANSS and BPRS, and 2) test whether psychometrically-informed scoring of PANSS/BPRS alters estimated antipsychotic effect sizes compared with conventional total-score analyses, and 3) to explore whether any changes differ across symptom domains and by sex.

**Method:**

We will conduct secondary analyses of anonymised individual participant data from randomized Phase II–IV schizophrenia trials accessed via secure data-sharing platforms (YODA Project and Vivli). Item-level PANSS/BPRS data will be harmonised at baseline and outcome. Psychometric models will be derived using confirmatory factor analysis, item response theory, and network analysis. Stability and invariance analyses will be conducted, examining covariates such as age and sex. Antipsychotic efficacy will be estimated using mixed-effects participant-level models, expressed as Cohen’s d standardised mean differences (SMDs) for both conventional and psychometrically-informed outcomes. The primary outcome will be the difference in SMDs between conventional and psychometrically-informed effect sizes, summarised across trials.

**Conclusions:**

This study will determine whether best practice psychometric refinement of existing measures meaningfully changes antipsychotic effect size estimates, informing outcome measurement choices and interpretation of trial results.

## Background

### Psychometric assessment and its relevance for estimating treatment effects in schizophrenia research

Multi-item psychometric scales are fundamental to clinical and psychiatric research and central to the assessment of symptom severity, treatment response and prognosis in schizophrenia. The Positive and Negative Syndrome Scale (PANSS ^1^) and the Brief Psychiatric Rating Scale (BPRS ^2^) are the most frequently used clinician-rated instruments to assess symptoms. Underpinning treatment guidelines and regulatory approval, ^3^ both scales are also widely used as primary outcome measures in randomised clinical trials (RCTs).

Over several decades, there has been substantial effort to evaluate the psychometric properties of both these instruments. In addition to approaches spanning classical test theory (e.g. confirmatory and exploratory factor analysis), more sophisticated statistical methods like Rasch-modelling and network analysis have been used in order to attempt to clarify the symptom structure, improve measurement precision or identify poorly performing items. ^4– 8^ This work has produced a large collection of evidence with differing or partially contradictory conclusions with respect to item functioning or scale structure and dimensionality. Most psychometric theories emphasise that validity is not an inherent property of an instrument, but rather that it depends on how well it measures the intended construct in a given population or context. ^9, 10^ Hence, when applied across different populations or interventions, continuous evaluation of psychometric scales using. ^11, 12^ However, it is not clear to what extent these continued psychometric refinements meaningfully improve conclusions drawn from studies using these instruments.

In principle, psychometrical optimization should increase statistical power by reducing measurement error, therefore, yielding more precise effect size. ^13, 14^ However, there is no consensus in the field that these expectations are empirically met, since a growing body of methodological literature shows that psychometrically refined scores are often. ^15, 16^ The claim of ‘improved measurement’ following psychometrical refinement therefore. ^11^ Current research in the field of depression has begun to fill this gap by linking psychometric analyses to the effect size estimation in randomised controlled trials (RCTs). Through combining individual participant data (IPD) from several RCTs, these investigations were able to compare treatment effects derived from psychometrically-informed models (from factor analytic, item response theory or network analysis models), to those with conventional total scoring. Results indicated that factor-analytical approaches could be increase estimated antidepressant treatment effects not universally improve outcome measurement the importance of careful interpretation of clinical relevance. Crucially, this emerging evidence reframes psychometrical optimization as a means to evaluate whether measurement choices substantially influence the estimation of treatment effects. From this perspective, it is no longer the question of how psychometric models best depict symptom structures, but whether conclusions drawn from such models meaningfully inform science, clinical practice or policy.

Despite continuous efforts in pharmacological development, reported effect sizes remain modest in schizophrenia trials. ^16, 17^ Although likely, it is still debatable whether shortcomings in the outcome measurements contribute to this pattern. ^6^ Despite extensive analysis of the psychometric properties of the PANSS and BPRS, no large-scale efforts have been made to assess whether psychometric refinements alter the estimated treatment effects in schizophrenia trials, nor whether such effects differ between demographic subgroups (e.g. sex) or symptom domains. This suggests the need for a systematic, large-scale assessment of psychometric approaches in schizophrenia research, explicitly examining their implications for effect size and outcome measurement.

The present protocol therefore describes how the EVIDENTIAL project ^18^ will address this gap in knowledge. This project will adopt similar methods to those described by Doyle and colleagues, ^19^ replicating and extending these to schizophrenia trials. The primary aim of the EVIDENTIAL project is to comprehensively examine the properties of the BPRS and PANSS to determine whether psychometric refinement might meaningfully alter schizophrenia symptom structure or performance, and more crucially, whether these changes might influence estimates of treatment efficacy examined in subsequent analyses. To achieve this, we have three objectives, corresponding to three research stages:

- To determine the best psychometric models for the PANSS and BPRS
- To test whether psychometrically-informed scoring of PANSS/BPRS alters estimated antipsychotic effect sizes compared with conventional total score analyses
- To explore whether any changes differ across symptom domains and by sex.

## Methods

### Study design

This study is a secondary IPD re-analysis of randomized controlled trials of antipsychotics in schizophrenia, schizoaffective or schizophreniform disorders. Item-level symptom ratings from the PANSS and BPRS will be collected and psychometrically analysed at baseline and outcome assessment. Reporting will follow STROBE ^20^ principles, and any deviations from the prespecified plan will be documented.

### Setting, participants and eligibility criteria

This study is open to randomized controlled multi-arm trials (Phase II-IV) comparing the effects of pharmacological treatments with antipsychotics to placebo (primary outcome) or any comparator (sensitivity analysis). Individual trial size is not an eligibility criterion. Eligible trials will include adult participants diagnosed with schizophrenia, schizoaffective or schizophreniform disorders. Trials must include PANSS and/or BPRS individual participant data at baseline and outcome. Studies meeting the criteria described in Table 1 have been requested from vivli.org (Vivli) and yoda.yale.edu (YODA Project). Requested studies from the YODA Project are solely sponsored by Johnson & Johnson. In addition, Vivli provides data from the following sponsors: Abbott, AbbVie, Janssen, Johnson & Johnson, Lilly, Otsuka. Previous data checks suggests that we should expect to obtain a pooled sample of ~31 000 participants from 77 studies provided on the Vivli platform ( Table 2).

**Table 1.** Inclusion/exclusion criteria for selecting trials and participant data.

Variable	Inclusion criteria
Study Type	Randomized controlled multi-arm trial
Data Type	Individual Participant Data
Phase	II, IIa, IIb, III, IIIa, IIIb, and IV
Population/Diagnostic Criteria	Participants with Schizophrenia Spectrum Disorders, aged 18 years and older
Treatment	Antipsychotic medication
Comparator	*Placebo* (to establish efficacy) *Active Comparator* (to establish baseline psychometric model)
Active Comparator	*Primary* (standard) *:* first-generation vs second-generation antipsychotics (optionally adding D2 partial agonists as a third category where feasible). *Exploratory:* receptor-based taxonomy as proposed by McCutcheon et al. ^21^
Outcome	Positive and Negative Syndrome Scale (PANSS) and/or Brief Psychiatric Rating Scale (BPRS)
Covariates	Sex (if available: Age)
Moderator Variable	Sex Stage of illness (sensitivity analysis)
Timing of Outcome Assessment	6 weeks, as per Younis et al. ^22^/ *4 weeks for sensitivity analysis*
	**Exclusion Criteria**
	All studies outside of the above parameters

**Table 2.** Expected Sample.

Database One:		https://yoda.yale.edu/ (YODA Project)
Number of studies requested	-	62
Number of studies by sponsor		
*Janssen*	-	26
*Johnson & Johnson*	-	36

Database Two:		www.vivli.org (Vivli)
Number of studies requested	-	77 (62 are overlapping with YODA)
Number of studies by sponsor		
*Abbott*	-	1
*AbbVie*	-	1
*Lilly*	-	9
*Janssen*	-	26
*Johnson & Johnson*	-	36
*Otsuka Pharmaceuticals*	-	4

### Outcome measures *:* PANSS and BPRS

The PANSS ^1^ is one of the most widely used clinician-rated outcome measures in schizophrenia research. It comprises 30 items, each rated on a 7-point anchored severity scale (1 = absent to 7 = extreme), which is commonly summarised as three symptom groupings: Positive (7 items), Negative (7 items), and General Psychopathology (16 items), together forming a total score ranging from 30 to 210. Despite its extensive use, heterogeneity in reported factor solutions and item functioning has prompted concerns about the PANSS’ psychometric robustness and its sensitivity to treatment-related change under some conditions. ^6^^,^^23^

The BPRS is another long-established clinician-rated scale frequently used in psychopharmacology research to quantify a broad range of psychiatric symptoms (e.g., thought disturbance, affective symptoms, activation, hostility). ^2, 24^ We will utilize the BPRS version comprising 18 items, each rated on a 7-point severity scale (1 = not present to 7 = extremely severe), producing total scores that typically range from 18 to 126. As with PANSS, published work indicates that factor structures and item performance can vary across samples and settings, which is relevant when BPRS totals are used as trial outcomes. ^7, 8, 25, 26^

### Statistical analysis plan

Analyses will be conducted within the secured environment of Vivli. Our approach will follow a two-stage workflow. First, we will use item-level PANSS and BPRS data to evaluate and select the best-fitting, state-of-the-art psychometric models of symptoms. Second, we will use the resulting psychometrically-informed scores to estimate antipsychotic treatment effects and compare these with effects derived from conventional PANSS/BPRS total scores, thereby quantifying whether and how psychometric modelling alters trial-level and pooled treatment effect estimates. We will follow a similar methodological approach as detailed in the publications provided by Doyle et al., ^19^ regarding the exploratory and confirmatory factor analyses (EFA, CFA) and Byrne et al., ^11, 13, 15^ regarding the item response theory framework (IRT) and network analytical approach (NA).

### Stage 1: Establishing optimal psychometric models of PANSS and BPRS

### Exploratory and Factor analytic approaches

Data will be randomly split into exploratory and confirmatory datasets, as recommended by Lubke and Luningham, ^27^ with stratification by treatment group and trial to ensure balanced representation. For stratification and subsequent subgroup/invariance checks, active antipsychotic arms will be coded using literature-standard classification (primary approach: first-generation vs second-generation antipsychotics; where feasible, a third category for D2 partial agonists may be considered). In exploratory analyses, we will additionally code active agents using McCutcheon and colleagues’ data-driven receptor-based taxonomy, ^21^ namely muscarinic (M2-M5) receptor antagonists; dopamine (D2) partial agonists and adrenergic antagonists; serotonergic and dopaminergic antagonists; and dopaminergic antagonists. We will ascertain factor models of schizophrenia for baseline (pre-treatment), combined outcome (post-treatment; 6 weeks) and separately for placebo and treatment groups. We will further evaluate measurement invariance across major arm categories (e.g., placebo vs active treatment and, where supported, across active treatment-arm classes) within the best-fitting baseline model(s).

Parallel analysis will be applied to the exploratory datasets to determine symptom dimensionality. ^28^ Next, the EFA will be performed, using oblique rotation to allow for correlated symptom domains, to examine the factor structure of the exploratory dataset. Models will be refined iteratively by evaluating factor loadings below 0.3 and/or communalities below 0.4, with poorly performing items removed where justified to achieve stable and interpretable solutions. ^29^

In the confirmatory dataset, CFA will be used to formally test candidate psychometric models identified during the exploratory phase. In addition to simple structures derived from EFA, established alternative models, including higher-order and bifactor representations, will be evaluated. ^30^ To select the optimal model of schizophrenia, the selection process will be guided by established absolute and relative fit indices, information criteria, and internal consistency metrics, including: the Root Mean Square Error of Approximation (RMSEA; < 0.08), Standardised Root Mean Square Residual (SRMR; < 0.08), Comparative Fit Index (CFI; > 0.95), Tucker-Lewis Index (TLI; > 0.95), Akaike Information Criterion (AIC), Bayesian Information Criterion (BIC), and McDonald’s Omega (acceptable >0.70), following established practices. ^31, 32^ Similar to the exploratory approach, confirmatory modelling will be performed for baseline and for the primary post-baseline outcomes. For outcome (post-baseline) measurement models, we will estimate the best-fitting model in the pooled sample and then test invariance across key arm categories (placebo vs active; and where feasible across active-arm classes as defined by the standard classification, with McCutcheon’s taxonomy explored in sensitivity analyses). We will retain the best-fitting baseline and post-baseline model(s) to derive factor scores for downstream treatment-effect estimation in Stage 2. ^33^

Measurement invariance, i.e. metric invariance (factor loadings are equal) and scalar invariance (intercepts are equal) across sex will be assessed, and differential item functioning examined by mean, standard error, standard deviation, factor loading scores and *r ^2^* values. When invariance is violated, psychometrically optimized models will be examined separated by sub-group (e.g., women and men).

The outcomes of these analyses will then be used for further evaluation and discussion (descriptively) on their differences or similarities in all models and datasets. This procedure will include model structure or item functioning (i.e. dropped items, item loadings on factor). Revised sum scores based on the retained items and weighted scores will then be computed from the best performing baseline and combined outcome model for each measure. These will be used for the stage 2 analysis described in the following sections.

### Item response theory approaches

Similar to the procedure described above, we will also use IRT approaches. ^34, 35^ In IRT, each item is treated as providing information about a latent trait, and items are evaluated by how strongly they discriminate between individuals at different levels of that trait (i.e., higher latent severity increases the probability of endorsing higher response categories). Within an exploratory dataset, we will use non-parametric IRT (Mokken scaling) to assess scalability, dimensionality, and item ordering, and to identify poorly discriminating items. Following recommendations by Meijer and Baneke, ^36^ we will evaluate solutions across increasing lower-bound scalability thresholds (c), beginning at c = 0.01 and then stepping by 0.05 (i.e., 0.05, 0.10, 0.15 … up to 0.60), selecting the most interpretable solution. Second, using the confirmatory dataset, we will fit parametric IRT graded response models to estimate item parameters (e.g. discrimination and category thresholds) to identify the best fitting model. Hence, we will compute revised sum scores based on the retained items and generate latent trait (weighted) scores for use in downstream analyses within stage 2. ^37^ A detailed description of the analysis principles for the IRT approach is outlined in Byrne’s publications. ^11, 13^

### Network analytic approaches

Network analysis is a more recent approach to psychometrics, which differs from traditional theories in that it does not assume that symptoms are caused by a single underlying latent trait. Instead, NA conceptualises symptoms as mutually interacting components, represented as a graph in which nodes are symptoms and edges reflect conditional associations between symptoms. ^38, 39^ This perspective has been proposed as a clinically intuitive complement to traditional models of psychopathology. Using best practice approaches, we will estimate empirical network structures separately for baseline (pooled across arms) and outcome data (separated by treatment arm). Network analysis will follow an exploratory graphical workflow, to identify the dimensional structure and detect redundant or weakly-performing items. As recommended by Christensen and Golino, ^40^ bootstrap-based stability assessment will be applied and empirical networks will be assessed in relation to the bootstrapped samples. Their work also suggests that replication metrics around the ~0.65–0.75 range mark the point at which variables begin to show reduced stability, providing a practical basis for identifying potentially unstable items. ^12, 40^ A key goal is to identify a network structure that is stable and configurally invariant across baseline and outcome (or treatment arms). If invariance cannot be achieved, we will proceed with the network solution with fewest/least consequential instabilities. From the final network solution, we will compute (i) revised sum scores based on the retained items, and (ii) weighted “net scores” derived from network loadings/strength. A detailed description of the analysis principles for the NA approach is outlined in Byrne’s publication. ^15^

### Stage 2: Does psychometric modelling matter? Modelling change and calculating effect sizes

In stage 2 we apply the psychometrically-derived outcomes from the stage 1 analysis to evaluate whether psychometric refinement alters estimated antipsychotic treatment effects compared to original total scores.

### Primary analyses: Continuous outcomes

For each instrument (PANSS and BPRS), outcome symptom severity will be modelled as a function of treatment assignment, adjusting for baseline symptom severity and accounting for clustering of participants within trials via random intercepts, as per best practice RCT modelling. ^41^ Detached from the psychometric modelling approach (CFA, IRT, NA) in Model 1 the overall effects of antipsychotics versus placebo, using conventional scoring (i.e., original raw scores) will be estimated. Model 2 comprises a set of related models that mirror Model 1, where raw scores are replaced with abbreviated sum scores based on the results of the psychometric analysis from stage 1. In Model 3 psychometrically-informed (weighted) scores, that are derived from the abbreviated models will be established. Model 2 and 3 will be calculated for each psychometric method separately (CFA, IRT, NA). Models 4, 5 and 6 will extend Models 1, 2a-c and 3a-c, by additionally adjusting for participant level covariates:

• Model 1 (conventional scoring): Outcome is the raw total score with treatment group and baseline score as covariates and a random intercept for trial.
• Model 2 (abbreviated total scores): Outcome is a simplified total score created after removing redundant items (as per relevant psychometric method) treatment group and corresponding baseline abbreviated score included as a covariates and a random intercept for trial.
  o Model 2a – factor analysis abbreviated total score
  o Model 2b – IRT abbreviated total score
  o Model 2c – NA abbreviated total score
• Model 3 (weighted psychometric scores): Outcome is a psychometrically derived score (e.g., factor score, IRT expected score, or net score) treatment group and corresponding baseline abbreviated score included as a covariates and a random intercept for trial.
  o Model 3a – factor analytic weighted scores
  o Model 3b – IRT weighted scores
  o Model 3c – NA weighted scores (net score)
• Model 4: Model 1 additionally adjusting for participant-level covariates (e.g. sex, age [where available])
• Model 5a-c: Model 2a-c additionally adjusting for the same participant-level covariates
• Model 6a-c: Model 3a-c additionally adjusting for the same participant-level covariates

Effect estimates from each model will be transformed into Cohen’s d to facilitate comparison across trials and scoring approaches. The primary effect of interest is the difference between SMDs derived from those derived from conventional scoring and psychometrically-informed outcomes (i.e. Model 1 vs Models 2a-c vs Model 3a-c, and Model 4 vs 5a-c vs Model 6a-c).

### Trial-level analyses

In addition to pooled IPD analyses, treatment effects will be estimated separately within each trial using both conventional and psychometrically-informed outcome scores. For each trial, change from baseline to outcome will be modelled adjusting for baseline severity, and trial-specific SMDs will be calculated for both scoring approaches. The difference between the psychometrically informed SMD and the conventional SMD will be computed for each trial and treated as the trial-level effect of interest. Potential moderators (e.g. sex) may be explored descriptively or within mixed models where data permits.

### Secondary analyses: Response

Secondary analyses will explore whether psychometric refinement meaningfully alters estimates binary outcomes such as response. Response will be defined using established PANSS or BPRS definition. ^42– 44^ Because binary outcomes may be sensitive to how thresholds map onto the underlying scale, these analyses will be treated as exploratory. The change in number of participants between psychometrically informed and original data will then be analysed using multilevel logistic regression models. Models will be fitted with and without adjustment for participant-level covariates.

Analyses will proceed in three steps:

1. Estimate baseline to outcome change in the proportion meeting response criteria within each arm, using both conventional and psychometrically-informed scoring.
2. Estimate treatment and comparator differences in response using conventional scoring and psychometrically-informed scoring in separate models.
3. Quantify the % difference between these two estimates (psychometrically-informed vs conventional) as the key comparison for secondary outcomes.

### Exploratory subgroup and sensitivity analyses

Where feasible and supported by data availability, we will explore whether psychometric models and treatment effects vary by sex. Measurement invariance across sex will be assessed within the optimal psychometric models using established procedures. Where invariance is violated, group-specific psychometric models will be estimated, and primary and secondary analyses repeated using sex-specific scores.

To further examine the robustness of findings to potentially important sources of between-trial and clinical heterogeneity, we will attempt to conduct additional exploratory sensitivity analyses:

• Endpoint timing (week 4 vs week 6): Given that acute antipsychotic trials commonly report outcomes around week 6, but earlier endpoints (e.g., week 4) may yield different conclusions. ^22^ Therefore, we will, where endpoint timing can be harmonised and sample size allows, repeat analyses stratifying trials by the closest available acute endpoint (week 4 vs week 6).
• Stage of illness: Where sufficiently detailed and harmonisable information is available, we will explore whether the best-fitting psychometric model(s), invariance results, and estimated treatment effects differ according to patients’ schizophrenia history (e.g., first-episode/early-phase versus multi-episode/chronic presentations, prior treatment exposure, or other trial-reported indicators of illness course).

These exploratory analyses are motivated by the clinical and methodological need to understand whether modelling choices or trial-design and population differences (such as endpoint timing or illness course) could materially influence inferences about treatment effects. All subgroup and sensitivity analyses will be clearly labelled exploratory, and interpretation will prioritise effect estimates and uncertainty, with transparent reporting of any constraints arising from limited sample sizes, inconsistent variable availability, or restricted harmonisability across trials, consistent with STROBE reporting principles.

### Software and reproducibility

All analyses will be conducted using reproducible statistical workflows in R and RStudio, with additional software used for meta-analytic synthesis where appropriate. Analysis scripts and documentation will be shared in open repositories where permitted, in line with FAIR principles and Open Science guidelines.

### Patient and Public involvement (PPI)

Patient and public involvement will be integrated to ensure that outcome interpretations and dissemination plans reflect the patient’s priorities and are communicated in accessible language. Therefore, we will establish a panel including people with lived experience of psychosis/schizophrenia recruited through a clinic network in Ireland. The panel will meet 3–4 times for approximately 60 minutes via online sessions supported by brief material. Participants will be remunerated as per institutional policy. PPI provided input will include structured discussions and written feedback in (i) plain-language summary, (ii) framing of “clinically meaningful change” for PANSS/BPRS on an individual level, (iii) planned subgroup analysis reporting and equity implications, and (iv) dissemination formats. Results will be reported according to the GRIPP2 short form checklist. ^45^

## Discussion

Advanced psychometric methods are widely advocated in the methodological literature as tools to improve measurement quality in clinical research. ^46, 47^ Yet, it remains uncertain whether they change what matters most – the estimated efficacy of interventions. This protocol is designed to test that practical question in schizophrenia research by quantifying whether psychometrically-informed scoring substantially alters antipsychotic treatment effect estimates.

Doyle et al. proposed, ^19^ and Byrne and colleagues subsequently demonstrated ^13^ that applying psychometric refinements can yield detectable differences in estimated antidepressant treatment effects, with the most consistent changes observed for factor-analytic approaches, while IRT- and network-based variants often produced little added value or inconsistent shifts in effects. The present project directly builds on this framework by translating it into schizophrenia trials, where measurement challenges and modest effects are long-standing concerns. Meta-analytic evidence from placebo-controlled antipsychotic drug trials have shown limited improvement across decades, suggesting that increasing placebo response is one plausible contributor to diminished effect sizes. ^17^ If placebo response grows (or baseline severity declines), detecting drug effects becomes more difficult, even when drugs remain efficacious, making it especially important that outcome measurement is as informative and low-noise as possible. Simultaneously, the interpretability of outcomes depends on the measurement properties of the instrument. A recent synthesis by Geck and colleagues concluded that the PANSS shows adequate performance regarding responsiveness and reliability, but has notable shortcomings in content validity and structural validity. ^6^ These limitations can weaken confidence in what total and subscale scores represent and how changes should be interpreted.

In this context, psychometric re-modelling using pooled IPD may help clarify which symptom structures replicate reliably, which items contribute meaningfully, and whether abbreviated scales will capture the same responsiveness to treatment or whether weighted scoring yields more accurate estimates of change. Importantly, we do not claim that psychometric refinement necessarily produces clinically meaningful change at the individual level. Rather, the planned analysis evaluates whether relatively modest differences in effect size (e.g., ~10–15% as observed in prior IPD psychometric work) are detectable and may improve the precision, interpretability, or comparability of trial effect estimates. Yet, they can still matter for meta-analytic synthesis, trial power, and the likelihood of detecting true treatment differences under challenging conditions.

### Limitations

Several limitations should be acknowledged. Our planned analysis relies on available trials within controlled-access repositories: Hence, data might vary regarding follow-up timing, inclusion criteria, and data completeness, which may influence both psychometric models and effect size estimates. Psychometric BPRS and PANSS modelling across heterogeneous trials may yield more than one statistically defensible solution. We will therefore prioritise transparency in model comparison and report repository-specific results. Psychometric refinement may improve measurement in some contexts, but not in others. Data user agreements with Vivli and YODA Project may limit sharing of IPD and restrict reproducibility to code/scripts and permitted metadata, rather than full data transparency.

## Ethics and consent

Ethical approval for the planned study was granted for the data collection by the Royal College of Surgeons in Ireland Research Ethics Committee (HREC Reference Number: REC202506033). Consent was not required for this study because no data were collected or analysed for this manuscript. The planned analyses will use secondary data, and consent for the use of participants’ data in future research beyond the original study was obtained by the respective study sponsors.

## Generative AI statement

The author(s) declared that Generative AI was used in the creation of this manuscript. During the preparation of this work authors used ChatGPT (GPT-4o mini, OpenAI) to refine the draft wording as well as a free online version of DeepL Write in order to cross-check the English phrasing and grammar. After using these tools, the authors reviewed and edited the content as needed and take full responsibility for the content of the published article.

## Data Availability

The data management plan and documentation describing the trials included in this study are openly available through Zenodo (v1.0; DOI: https://doi.org/10.5281/zenodo.19557064) and the Open Science Framework (v1.0; https://osf.io/xf6ac/files/6nb8g). Access to the underlying study data was obtained via Vivli ( www.vivli.org) and the YODA Project ( https://yoda.yale.edu/). The IPD used are not openly shareable because they comprise anonymised personal data and are subject to legal, ethical, and contractual restrictions, including GDPR requirements, consent restrictions, data use agreements, and sponsor or platform terms. Access to these data may be requested through Vivli and the YODA Project, subject to their respective governance and access requirements. Openly shareable materials will be provided via Zenodo and OSF under an open licence. These materials include non-disclosive derived outputs, such as aggregate trial-level estimates, psychometric parameters where permitted, model fit indices, analysis code, documentation, and synthetic or example data. These resources provide transparency regarding the trial selection and the analyses underlying the findings reported in this article.
