# Supplementary material for: Applying advanced psychometrics to antipsychotic treatment trials: A protocol comparing effect estimates
Source: Open Res Eur. 2026 Jun 17;6:211. [Version 1] doi: 10.12688/openreseurope.23623.1 (PMC13560577; doi:10.12688/openreseurope.23623.1)
Supplement: Peer review history

# Reviewer report: Reviewer response for version 1

**DOI:** 10.21956/openreseurope.25577.r78518

**Published:** 2026-08-28

**Authors:** Felice Iasevoli

**Affiliations:** 1 University School of Medicine of Naples Federico II, Naples, Italy

This is a thoughtful and potentially valuable protocol addressing an important methodological question: whether psychometrically informed scoring of PANSS and BPRS meaningfully changes estimated antipsychotic treatment effects. The planned use of large-scale individual participant data, together with factor analytic, item-response and network approaches, is a major strength. I also appreciate the attempt to move beyond purely psychometric model fit and ask whether alternative scoring approaches materially influence treatment-effect estimation. I consider this a strong and worthwhile project. My main concern is not the use of industry-sponsored data per se, but whether psychometric models derived from a highly selected RCT ecosystem will generalize beyond that context, and whether a larger treatment-effect estimate will be interpreted too readily as evidence of better measurement. The study would be substantially strengthened by clearer separation between model development and out-of-sample treatment-effect evaluation, ideally at the trial level, together with a more explicit definition of what constitutes psychometric improvement and a stronger justification for network-derived weighting. These issues appear addressable within the proposed dataset and would, in my view, materially improve both the methodological rigor and the interpretability of the eventual findings. I describe these concerns below in more details:

 The available datasets appear to come almost exclusively from pharmaceutical industry-sponsored trials accessed through YODA and Vivli. I do not regard industry sponsorship itself as a fatal limitation, particularly because the present study re-analyses randomized IPD independently of the original sponsor conclusions. However, I think the more important issue is the measurement context represented by these trials. Large industry-sponsored schizophrenia RCTs are highly selected environments, often involving restrictive eligibility criteria, standardized rater training, run-in or stabilization procedures, specific baseline severity requirements, and a limited range of antipsychotic agents.

 Psychometric models derived from such data may therefore characterize PANSS/BPRS performance under the conditions of regulatory or commercial RCTs rather than necessarily generalizing to schizophrenia populations or antipsychotic treatment studies more broadly. Could the authors clarify the intended target population to which the final psychometric models are meant to generalize? I would also encourage sensitivity analyses examining whether model structure and treatment-effect changes differ according to sponsor, trial design characteristics, and, where possible, medication class. If investigator-initiated trials cannot practically be included, this limitation should be framed primarily in terms of transportability rather than sponsorship bias alone.

 The protocol appropriately separates exploratory and confirmatory datasets for EFA/CFA development. However, the main scientific question is not simply whether a psychometric structure can be replicated, but whether a scoring rule derived from that structure changes treatment-effect estimation in data not used to construct the rule. Could the authors clarify whether treatment-effect comparisons will be performed in data that are independent from those used to derive and select the psychometric models? With an expected sample of approximately 31,000 participants across many trials, a stronger validation strategy seems feasible. In particular, I wonder whether trial-level cross-validation could be considered. For example, psychometric scoring models could be derived in all but one trial and then applied to the held-out trial, iterating across studies. This would directly test whether psychometric refinement alters drug–comparator separation in a previously unseen RCT, which seems closer to the intended practical application than participant-level random splitting alone.

 A central conceptual issue is that a larger treatment effect is not necessarily equivalent to better psychometric measurement. For example, a reweighted PANSS score could yield a larger drug–placebo standardized mean difference because it places greater weight on symptoms that are particularly treatment-responsive, without necessarily providing a more valid or more comprehensive measure of overall schizophrenia symptom severity. I therefore think the protocol should prespecify more clearly what will count as successful psychometric improvement. Is the primary criterion larger drug–placebo separation, greater statistical precision, lower measurement error, reduced between-trial heterogeneity, improved construct validity, improved responsiveness, or some combination of these? The interpretation of the study will depend heavily on this distinction.

 I would encourage the authors to avoid equating a larger SMD per se with superior measurement and to define in advance how statistical improvement, psychometric validity and clinical relevance will be distinguished.  The factor analytic and IRT approaches have a relatively clear rationale for deriving weighted or latent severity scores. I am less certain about the conceptual basis for translating network-analytic quantities into a single weighted treatment-outcome score. In network models, node strength or centrality reflects conditional relations among symptoms and does not necessarily imply that a symptom is more reliable, more informative regarding overall severity, or more appropriate to weight more heavily in a treatment outcome. Could the authors provide a stronger theoretical and statistical justification for deriving “net scores” from network loadings/strength? In particular, why should network centrality be expected to provide valid item weights for estimating treatment effects? This seems important because the network approach is conceptually distinct from latent-variable models and should not automatically be interpreted as an alternative method for estimating a unidimensional severity score.  

 The primary participant-level models include a random intercept for trial. Given the expected number and heterogeneity of included RCTs, I wonder whether this is sufficient. Treatment effects are likely to vary across trials because of differences in population, baseline severity, duration, formulation, medication, run-in procedures, placebo response and other design characteristics. I therefore think the analysis plan should clarify whether treatment-by-trial heterogeneity will be explicitly modelled, for example through random treatment slopes, or whether a two-stage IPD meta-analytic approach with trial-specific treatment effects and random-effects synthesis will be the principal inferential framework. This issue seems particularly relevant because the main quantity of interest is the difference between conventional and psychometrically informed treatment effects. It would be informative not only to estimate the average difference, but also to determine whether that difference is consistent across trials or highly context-dependent.  

 I agree with the concern already raised by another reviewer regarding the derivation of separate baseline and post-treatment measurement models. If the measurement structure or item parameters differ across time, baseline and outcome scores may not represent the same construct on the same metric. Could the authors therefore clarify whether longitudinal measurement invariance will be formally assessed before interpreting score differences or treatment effects based on psychometrically informed scoring? In my view, this is particularly important for any model in which baseline-adjusted outcome scores are interpreted as reflecting treatment-related change.

 The protocol contains a large number of psychometric approaches, alternative scores, subgroup analyses, drug classifications, outcome definitions and sensitivity analyses. This richness is valuable, but it also creates considerable analytical flexibility. I would therefore encourage the authors to define a clear hierarchy of primary psychometric model; primary scoring comparison; primary treatment-effect contrast; confirmatory versus exploratory analyses. The protocol should also state how multiplicity and selective interpretation across CFA, IRT, network models, abbreviated scores, weighted scores and subgroup analyses will be handled.

Is the study design appropriate for the research question?

Yes

Is the rationale for, and objectives of, the study clearly described?

Yes

Are sufficient details of the methods provided to allow replication by others?

Yes

Are the datasets clearly presented in a useable and accessible format?

Not applicable

Reviewer Expertise:

NA

I confirm that I have read this submission and believe that I have an appropriate level of expertise to confirm that it is of an acceptable scientific standard, however I have significant reservations, as outlined above.

# Reviewer report: Reviewer response for version 1

**DOI:** 10.21956/openreseurope.25577.r78521

**Published:** 2026-08-14

**Authors:** Yaoyao Sun

**Affiliations:** 1 Peking University Sixth Hospital, Peking University Institute of Mental Health, Beijing, China

Thanks for giving me the opportunity to review the interesting manuscript. This protocol addresses an important and clinically relevant methodological question: whether psychometrically informed scoring of the PANSS and BPRS can meaningfully alter estimates of antipsychotic treatment effects in schizophrenia trials. The proposed use of pooled individual participant data and multiple psychometric approaches, including factor analysis, item response theory, and network analysis, is promising. I hope several issues can be clarified:

 1. The concept of “psychometric optimization” remains insufficiently defined. I would suggest the authors to clarify exactly what is being optimized. For example, item selection, symptom structure, weighting, measurement precision, or treatment-effect estimation and what changes are expected. Would the proposed methods increase drug–placebo separation, reduce between-trial heterogeneity, improve precision, or reduce sensitivity to placebo response? 

 2. It is unclear why investigator-initiated trials (IITs) are not included. High-quality IITs may provide important evidence and improve the generalizability of the findings. Conversely, relying mainly on pharmaceutical industry-sponsored trials may introduce sponsorship-related bias in study design, participant selection, and treatment-effect estimation. The authors should justify this restriction and, where possible, include sponsor type as a moderator or sensitivity analysis.

 3. The Introduction acknowledges that psychometric validity may vary across populations and contexts, but cultural differences are not considered in the Methods. The authors should consider geographical region, language version, or cultural background in measurement-invariance or sensitivity analyses, where sufficient data are available.

 4. The protocol should more clearly separate model development from treatment-effect estimation. Developing psychometric models and estimating treatment effects in the same data may lead to overfitting and inflated differences from conventional scoring. 

 5. The treatment effect reflects change from baseline to follow-up, but the protocol appears to develop separate baseline and post-treatment measurement models. If the factor structure or item parameters differ across time points, the resulting scores may not be directly comparable. Longitudinal measurement invariance should therefore be assessed before interpreting score changes as treatment effects.

 6. The protocol includes many psychometric models, scoring approaches, subgroup analyses, and sensitivity analyses. The authors should clearly identify the primary model and primary comparison and describe how multiplicity and selective interpretation will be addressed.

 7. Clinical interpretability should be considered alongside statistical improvement. Even if psychometrically informed scoring produces a larger effect size, it remains uncertain whether the difference is clinically meaningful. The authors should prespecify how the magnitude and clinical relevance of any observed change will be evaluated.

Is the study design appropriate for the research question?

Yes

Is the rationale for, and objectives of, the study clearly described?

Yes

Are sufficient details of the methods provided to allow replication by others?

Yes

Are the datasets clearly presented in a useable and accessible format?

Yes

Reviewer Expertise:

biological psychiatry, clinical psychiatry, individualized treatment, pharmacogenomics, genome-wide association analysis

I confirm that I have read this submission and believe that I have an appropriate level of expertise to confirm that it is of an acceptable scientific standard.

# Reviewer report: Reviewer response for version 1

**DOI:** 10.21956/openreseurope.25577.r77697

**Published:** 2026-08-11

**Authors:** Robin Emsley

**Affiliations:** 1 Stellenbosch University, Stellenbosch, Western Cape, South Africa

I have conducted this review from a clinical researcher viewpoint rather than a statistical perspective.

 The manuscript describes a protocol aiming to assess the best psychometric models for the PANSS and BPRS scales for symptom assessment and treatment response in schizophrenia. The topic is of high importance. There is an enormous literature on studies of symptom severity and treatment outcome in schizophrenia, with many uncertainties remaining.  The planned study has been carefully composed and  includes a large sample of individuals from well characterised treatment outcome RCTs.

1. It is important to put the history of schizophrenia of rating scales into context: The PANSS was derived from BPRS, with additional items from the Psychopathology Rating Schedule added to provide more comprehensive assessment, particularly negative symptoms. The PANSS has become overwhelmingly the instrument of choice to assess symptom severity and changes over time, largely because of its relative clarity and ease of use in clinical settings. The 30 selected items were originally grouped into 3 subscales, positive, negative and general psychopathology, based largely on clinicians’ judgement. However, subsequent factor analyses indicated many of these items loaded with different domains, and some items loaded inconsistently across studies. Numerous FA studies followed, mostly favouring a five-factor solution. It should be kept in mind that criteria to define factors are somewhat arbitrary and methodology varied across studies. Many of these studies propose specific domain groupings and exclusion of certain items, basing their findings on extensive analyses. Much of what is published may have addressed the aims of this study, at least in part. It would be important for the present study to carefully review the current literature and define what is established as well as the uncertainties, and to clarify how this study will advance our understanding of the topic, rather than this being yet another analysis of the PANSS, just with a different statistical approach.
2. Multiple demographic and clinical variables will need to be carefully considered in the analyses. Also, medication dose and degree of adherence may significantly influence outcomes.
3. The studies that are planned for inclusion will overwhelmingly involve participants treated with risperidone of its active metabolite, paliperidone. While the advantage here is that it reduces possible confounding effects of differential efficacy effects of antipsychotics, it limits generalisability of these findings to people treated with other antipsychotics. So, for example, risperidone and paliperidone are more likely than some of the other antipsychotics to produce extrapyramidal symptoms that have been shown to effect some PANSS item scores.
4. Another consideration is that many of the RCTs (particularly those with long acting injectable formulations) have an initial run in treatment period to stabilise participants before baseline assessment. Analyses of treatment response in these studies will show much less effect than those assessing acutely psychotic individuals at baseline.
5. One of the major problems with PANSS is that scoring has been inconsistently applied regarding absence of symptoms. On the item scale of 1 to 7 a score of 1 indicates absence of a symptom, giving an absolute minimum total score of 30. Some studies correct for this in the analyses, while many don’t. This has a particularly significant effect on outcomes that are reported as percentage reductions.
6. Minor comments:
  1. Paragraph 3 in Background: There are several sentences where words or phrases are omitted.
  2. Methods: Top of p 4: add *selected* before randomised controlled trials.

Is the study design appropriate for the research question?

Yes

Is the rationale for, and objectives of, the study clearly described?

Yes

Are sufficient details of the methods provided to allow replication by others?

Yes

Are the datasets clearly presented in a useable and accessible format?

Not applicable

Reviewer Expertise:

Clinical and treatment aspects of schizophrenia

I confirm that I have read this submission and believe that I have an appropriate level of expertise to confirm that it is of an acceptable scientific standard, however I have significant reservations, as outlined above.
